# Abstracts and Gleanings

**Published:** 1880-02-20

**Authors:** 


### ABSTRACTS AND GLEANINGS.

**Gun-Shot Wound of Uterus—Bullet Traversing Six Months' Fœtus—Recovery of Patient in Four Weeks.**— We extract the following from a case reported by Geo. A. B. Hays, M.D., in the New Orleans Medical and Surgical Journal:

The ball (one from the same cartridge box weighed 136 grains) had penetrated the abdominal cavity at the left side, about two inches diagonally in front and above the anterior superior spinous process of the ileum, ranging upwards—confirming the statement that it had first struck the ground, the shot having been fired from on horseback—and had lodged within the abdominal cavity.

There had been but very little hemorrhage externally at the first, and I found a portion of omentum an inch in length protruding from the wound, completely plugging it. The woman had not menstruated since December, and stated that she was six months advanced in pregnancy. It was evident the abdominal wall was cut through, and furthermore, if the projectile had sufficient velocity at the moment of impact, the uterus and contents would be involved in the injury, but the distance from which the shot was fired, and the fact of the ball first striking the ground, prevented a positive diagnosis at the moment, probing of course being inadmissable. I reduced the protruded omentum and turned the woman upon her left side, in order to allow drainage in the event of internal hemorrhage or effused liquor amnii in the abdominal cavity. Prescribed full doses of sulph. morphia, and ordered large, warm linseed poultices, abundantly saturated with laud anum, to be constantly applied to the abdomen.

June 21st. Visited the patient at 10 a. m. She had well marked labor pains which had begun to come on about sunrise, as stated by the nurse. She had rested badly all night—scarcely slept at all. Entire abdomen extremely tender and distended. Could not bear light percussion. During a pain would by an effort of will control and arrest the contraction of the abdominal muscles, thereby throwing all the work upon the uterus. Shortly after 11 a. m., the contractions being stimulated by fluid extract ergot, the fœtus, placenta and membranes were expelled simultaneously with a very slight gush of waters when the membranes were ruptured. Some coagula escaped with the fœtus. After delivery the uterus contracted beautifully, not more than two ounces of blood being lost. Administered alcoholic stimulants and beef-tea, the patient being very much exhausted. Continued the poultices and fastened the bandage over them. Examined the fœtus for the pistol ball and found it had penetrated beneath the left scapula, ranged diagonally through the trunk a distance of about three inches, and made exit in the right hip. Careful search could not find the ball either in the placenta or among the coagula, and I was forced to the conclusion that it had entirely traversed the uterus, and as it could not be felt externally on the right side, that its course had been arrested just as it attained the inner surface of the abdominal parietes on that side. The child was a female, ten inches in length, well developed, nails formed, eyelids adherent. Evidently a six or six-and-a-half months fœtus.

Puerperal fever set in, accompanied by peritonitis. Opium, quinine and calomel were the remedial agents principally relied upon, and the poultices before mentioned were continued until recovery. Laxative enemas were sometimes resorted to. For the first few days of her illness I had no expectation of her recovery, and in fact more than once carried with me on my visit the necessary instruments to make an autopsy, expecting to find her moribund. In my treatment I was actuated greatly by the motto *Dum anima est, spes est,* and placed a deal of reliance upon the *vis medicatrix naturæ.*

From the 27th of June, her general condition underwent a change for the better and she steadily continued to improve. The opium, quinine and calomel were persisted in until her gums became touched, when the latter was discontinued, and a solution of chlorate of potash used as a mouth-wash. A strong camphor ointment relieved the inflamed breasts. She had very little lochial discharge, it lasting only two or three days. There was no drainage from the external wound at all, and it was closed with a strip of plaster and subsequently, when it suppurated a little, dressed with oxide of zinc ointment.

July 17th, she began menstruating with but little pain or discomfort; she ceased on the 19th, and the following day, July 20th, just one month from the date of the injury, she was dismissed well. It is proper to state here that the manager of the plantation kept an experienced nurse at the bedside day and night, and kept the patient supplied with everything necessary for her welfare, which undoubtedly contributed very greatly towards her reccvery. She is now, August 9th, walking about, feels well, sleeps well, has the appetite of a tramp, and suffers no inconvenience from the presence of the ball in her internal economy.

It would be interesting to know exactly by what physiological process the liquor amnii that escaped into the cavity of the abdomen became so readily absorbed, while the entire cavity was in such an altered and abnormal condition.

**External Urethrotomy by Wheelhouse's Method.**—An external examination of the perinæum revealed the position of the stricture. A scar of an abscess remained, and there was a thick fibrous cord running in the course of the urethra for about an inch. An exploration of the canal corroborated this. I then endeavored to reach the bladder through the stricture, and after a tedious but careful trial I failed. On this occasion, and on several others, I used a great variety of instruments, including caoutchouc and whalebone filiform bougies, which had served me well in other difficulties, but I could not find the narrow opening, and, on consultation with my colleagues, I determined to divide the stricture from the perinæum. In the *Dublin Journal of Medical Science* for December, 1876, p. 489, in my Half-yearly Report on Surgery, I had drawn attention to an operation devised for such cases by Mr. Wheelhouse, the distinguished surgeon, of Leeds. He had had much testimony in its favor, and I thought the proceeding so hopeful, and had seen such good results from it in a case subsequently operated on by Mr. Thornley Stoker, that I determined to adopt it. The details of the operation, as given by the author, are to be found in the *British Medical Journal*, 1869–70, and June 24th, 1876, and in part of this Journal, as mentioned.

The instruments required are—a special staff, an ordinary scalpel, two pairs of straight-bladed forceps, nibbed at the points, a well-grooved probe-pointed director, Teale's probe gorget, a straight probe-pointed bistoury, and a short silver catheter with elastic tube attached.

The patient, who suffered much from occasional rigors, was prepared for the operation by a course of tonic treatment, in which quinine was predominant. Having been brought into the theatre, he was placed in the lithotomy position, and secured as for that operation. I now introduced the staff of Mr. Wheelhouse. This staff is grooved upon one side to within half an inch of the end, and the extremity is a button-like hook, which projects very slightly on the opposite side. The instrument was introduced with the groove towards the perinæal surface, and was pressed firmly but gently against the stricture. It could be easily felt through the soft parts, and a few incisions only were necessary to reach the urethra. Then I passed the point of the knife into the groove, dividing the healthy part of the canal for about an inch. Care must be taken that the incision is made in the groove only, and not upon the point, "so as certainly to secure a quarter of an inch of healthy tube immediately in front of the stricture." This having been done, the edges of the urethral flaps were seized by forceps and held aside. The next step was to change the position of the staff. It was slowly withdrawn, until its extremity appeared through the wound. It was then turned completely round, so that the button-like hook looked towards me, and the groove towards the pubes. Continuing the withdrawal, the "hook" caught in the upper edge of the wound, in the urethra. Thus the parts were held open at three points.

We were now face to face with the stricture, looking from healthy to diseased urethra. Here began the difficulty of the operation, for I had to find the opening, which undoubtedly existed. There were no granulations in front of the stricture; but it looked a dense wall, through which we were not likely to find a passage. Taking the director, I made a prolonged search with it for the opening, and I was delighted to find it at last. The instrument was, however, too large, and was passed in with difficulty. By carefully dividing the cicatricial tissue upon it, I succeeded in reaching the bladder, although this proceeding took a good deal of time. Then the director was turned, so that the groove was turned towards the anus. Passing a knife along the groove, the whole stricture was completely divided, and the urine escaped with a gush. The next step was to introduce a catheter—always a great difficulty with perinæal operations. To effect this the probe point of Teale's gorget was introduced into the groove of the director and gently pushed along this into the bladder. The director was then removed, and we had an unyielding channel along which we could pass a catheter. A gum-elastic instrument was introduced through the meatus, and reaching the gorget, was conducted by it into the bladder.

The patient was removed to bed, and the urine was allowed to drain away through an India tube. He quickly rallied from the operation.

His evening temperature was 99·8°—the highest range reached—falling next morning to 98·4°.

On the fourth day I removed the catheter, and for some days afterwards I passed a silver instrument every morning. The wound gradually closed, and had quite healed in about three weeks. No bad symptoms supervened. The patient's bladder slowly recovered its tone, and he was able to retain water for four hours, when he left the hospital, exactly two months after his admission. He had been up for a month, but I kept him in the hospital in order to observe if there was any tendency of the stricture to return. When the catheter was not used for a week this tendency developed, but when he left the canal easily admitted a No. 12 catheter, and I impressed on him the need of having and instrument passed at short intervals.—*Haf-Year Compendium.*

**Permanganate of Potassa in Chronic Otorrhœa.**—In the *Buffalo Medical and Surgical Journal*, August, 1879, Dr. L. Howe records fifty-three individuals thus treated. Several others were noted, which would have been included in the list, but for the fact that the records were not complete, or that the patients were lost sight of before any definite conclusions could be arrived at.

The ages of the patients ranged from two to forty-four years, and the disease had lasted, with greater or less variation, from three weeks to twenty-two years. In several instances where the otorrhœa followed a primary acute otitis media purulenta, it was not easy to say when the discharge had passed into a condition to deserve the term "chronic." I have, therefore, excluded all cases in which the disease has not lasted at least three weeks, or which have still presented those symptoms of pain, etc., which would entitle them to the term "acute."

Concerning the etiology of these cases, there was about the same variety that one would expect in a series chosen at random. Scarlatina furnished its usually large quota of almost half—more exactly, twenty-one. Seven followed primary acute otorrhœa. Two resulted from meosles, and a number were dependent upon causes which it was impossible to ascertain.

The principal treatment prescribed in all of these, was the use of a solution of permanganate of potassa in water. In the milder cases two grains to the ounce was found to be sufficient, but if more severe, or of very long duration, the strength was increased to four, six or even eight grains to the ounce.

The patient was simply instructed to syringe out any accumulated secretion with tepid water, and then pour a few drops of the fluid into the ear. This was allowed to remain five or ten minutes, if it produced no smarting or burning sensation. If decided inconvenience followed, however, it was washed out sooner. When the discharge was at all abundant, this was repeated twice daily, and if very profuse, of course the ear was kept clean by more frequent washings with water alone. Whenever polypi developed, as was the case in three instances, or any other complications presented, they were treated by the usual methods.

A sufficient time has now elapsed to count, with considerable certainty, on the effects produced; and, as a result, I find: of the fiftythree cases forty have entirely recovered, in an average time of thirty-eight days. In six the discharge recurs at intervals; in four it is continued, but lessened. as to quantity and the fœtid character of the odor; in three it still persists.

Now, comparing this treatment with those methods usually placed foremost in the text books, I find the results are decidedly in its favor. In seven of these cases, nitrate of silver had been used after the manner recommended by Schwartz, and although an improvement was manifest in most of them, the progress was not as rapid as under the use of permanganate of potassa. Moreover, in ten other cases treated with nitrate of silver, the discharge was arrested in six, after an average of fifty-one days; two persisted in the treatment about six months and then disappeared; while concerning two, the record is imperfect. In three of the cases reported, I began by using sulphate of copper, but after an average of six days changed to the permanganate of potassa, and improvement followed quite as rapidly, to say the least, as before. Again, in five other cases treated with sulphate of copper alone, I find, on the average, the discharge continued for sixty-three days. Sulphate of zinc, alum and tannic acid, alcohol, carbolic acid and other remedies have been used in occasional instances, but the record concerning them is not sufficiently definite to warrant any exact statement.—*Half-Yeat Compendium.*

**Nitrous Oxide Gas.**—The administration of the gas is for its stimulating effects, and taken with a greater or less proportion of air, it produces a species of exhilaration with very little subsequent reaction, the primary excitement being akin to that which commonly follows the use of champagne or any sparkling dry wine. There is a feeling of lightness and buoyancy, the individual becomes loquacious, while the heart's action is increased, and there is a momentary dizziness. The administration of the gas should not produce other effects, for intoxication and anæsthesia imply a step beyond stimulation, and therefore should be avoided. If the fingers grow numb, or if any object upon which the patient fixes his gaze becomes blurred, then should the use of the gas be discontinued.

From the condition of exhilaration described the patient recovers, and for the rest of the day feels decidedly brighter and more active. If he be depressed before the gas is given, or suffers from an attack of "the blues," the effect is much more marked. Patients who have placed themselves under our care, who have at first been dejected, who have worried about their absence from home, or about their children, or business, have in a few days become entirely accustomed to their new surroundings, and have indulged in amusements and pursuits they never were able to enjoy. It virtually takes the place of alcoholic stimulation, without depression, and for this reason we suggest its use in the treatment of alcoholism, especially in the early stages of delirium tremens. As a remedy in insomnia it has no equal, and when *not* given just before bedtime, but in the morning or middle of the day, it, as a rule, procures refreshing sleep, even for those who have relied entirely upon anodynes.

In cases of so-called "spinal irritation," or in cases of "nervous prostration," in women who have no discoverable uterine disease, we have good reason to think it acts admirably, as it also does in hysteria, or the form of nervous derangement bordering on melancholia. When the circulation is defective, and when there is lividity of the hands, constipation, slow pulse, and rough, scurvy, mud-colored skin, it in a short time greatly betters the condition of the patient. It, so far as we have seen, favors the assimilation of food, hastens the action of iron, and increases functional activity. Appetite and sleep, and disposition to take exercise, are all improved.

The remedy has been used much in its compressed or liquid form by quacks, under the names "compound oxygen" or "liquid oxygen," and even in such hands has cured many persons who have gone the rounds of the legitimate profession.

It is not indicated in sthenic affections, or when there is a tendency to cerebral congestion or excitement, but rather for the irritability of exhaustion. It therefore, we have found, increases the trouble in vigorous, full-blooded subjects. The gas should be used well diluted with air, which may be accomplished by opening the valve near the mouth in the instrument provided for the purpose. Daily inhalations of not less than twenty gallons of gas should be used, the amount of air taken as well into the lungs being sufficient to prevent intoxication.

We are now engaged in experiments with nitrous oxide in the treatment of the chronic insane, and will publish our observations at another time. At present we deem it our duty to suggest to the profession the employment of a remedy heretofore used only for surgical purposes, which, in its new method of employment, promises a great deal.—*Medical Record.*

**Belladonna in Obstinate Constipation.**—In a case of typhoid fever recently in the wards, said Dr. Costa in the Mich. Med. News, constipation became so obstinate at the end of the disease, that I found it necessary to do something for it. It did not seem proper to resort to cathartics with the possibility of Peyer's patches being still unhealed. So I was forced to look elsewhere, and hit upon the following expedient. Every night before the patient went to bed he took a tablespoonful of sweet oil, and thrice daily after meals, he was given gtt. j of the fluid extract of belladonna in ʒ i of the compound tincture of cinchona (Huxham's). The effects of this treatment were admirable. In a day or two he had a normal passage every morning. Then, in order to see if this might not be due solely to the sweet oil, I left orders that the oil should be omitted for several nights, but the bowels were still regular. There was no question but that it was largely the belladonna.

**Compressed Nitrous Oxide as an Anæsthetic.**—Foreign journals inform us that M. Paul Bert's new method for producing anæsthesia—nitrous oxide used under pressure—has been introduced into the Paris hospitals. Last month, M. Leon Labbe performed seven surgical operations, of which the duration varied from five to thirty-two minutes, in the movable chamber put up at the Lariboisiere Hospital by Dr. Fontaine, for the surgical and medical employment of compressed air. As in the operations already performed at the medico pneumatic establishment in the Rue Chateaudun by M. Pean, the success of this new anæsthetic method was complete.—*Medical Reporter.*

**How to Apply the Hot Water Vaginal Douche.**—In the Chicago Medical Gazette, Dr. E. C. Dudley says:

The following is designed to impress the importance of strict observance of detail in the application of the douche, since in no other manner will its good effects be realized:

| *Ordinary Method of Application.* | *Proper Method of Application.* |
| --- | --- |
| I. Ordinarily the douche is applied with the patient in the sitting posture, so that the injected water cannot fill the vagina and bathe the cervix uteri, but on the contrary, returns along the tube of the syringe as fast as it flows in. | I. It should invariably be given with the patient lying on the back, with the shoulders low, the knees drawn up and the hips elevated on a bed pan, so that the outlet of the vagina may be above every part of it. Then the vagina will be kept continually overflowing while the douche is being given. |
| II. The patient is seldom impressed with the importance of regularity in its administration. | II. It should be given at least twice every day, morning and evening, and generally the length of each application should not be less than twenty minutes. |
| III. The temperature is ordinarily not specified or heeded. | III. The temperature should be as high as the patient can endure without distress. It may be increased from day to day, from 100° or 105° to 115° or 120° Fahr. |
| IV. Ordinarily the patient abandons its use after a short time. | IV. Its use, in the majority of cases, should be continued for months, at least, and sometimes for two or three years. Perseverance is of prime importance. |

The sitting posture is especially objectionable, for another reason: it favors pelvic congestion by force of gravity, while the dorsal position utilizes this force during the application of the douche.

A satisfactory substitute for the bed pan may be made as follows: Place two chairs at the side of an ordinary bed, with space enough between them to admit the lower bucket; place a large pillow at the extreme side of the bed nearest the chairs spread an ordinary rubber sheet over the pillow, so that one end of the sheet may fall into the bucket below, in the form of a trough. The douche may then be given with the patient's hips resting on the pillow and with one foot on each chair; the water will then find its way along the rubber trough into the bucket below.—*Medical and Surgical Reporter.*

**Acute Orchitis.**—Inflammation of the testes is a very distressing affection, whether traumatic or idiopathic in its origin; and any treatment that promises relief is worthy of consideration. The following cases will indicate a treatment that has proved highly satisfactory in my hands.

*Case* 1.—A. C., aged 45, had an attack of venereal urethritis, and attempted to treat himself by using some very strong injection, which he forced down the whole length of the urethra. This was followed by considerable irritation of the neck of the bladder, which kept up a continuous spasmodic action of the sphincter and a frequent desire to urinate all the next day. On the second morning he had severe pains in the left testis, and before night it was considerably swollen and intensely painful.

I was called in about noon of the third day and found the patient in a high fever and suffering greatly. I immediately ordered .6 grams (10 drops) of the green-root fluid extract of gelsemium, to be repeated every half hour till 4 grams (1 drachm) should be taken.

I also ordered the following :

℞ Ammoniæ muriatis.................................... ʒi

Phytolaccæ dec. fl. ext. (green-root)......................

Rect. spts............................................aa ℥i

Aquæ, q. s. ad.......... ................................. ℥ viij. M

Sig : Apply to the part with a cloth, and renew every hour.

The testes were also well supported by means of a handkerchief passed under them and fastened to the shirt over the pubis.

The gelsemium was given with the view of controlling the fever and relieving the pain, both of which it accomplished before the 4 grams had been taken. This was repeated in 3-gram doses whenever any fever or pain began to develop. The local application was also continued for two days, when the inflammation had entirely subsided, and in about a week's time the enlargement had almost disappeared.

*Case 2.*—G. R., aged 28, was struck on the right testis with a hard ball. Inflammation of a severe character at once set in, and had continued about a week before I saw the case. Then the testis was as large as a good-sized cocoanut, and the pain so severe that he had not slept for five days.

I ordered the same treatment as described above, and after the fourth dose of the gelsemium, the patient was sound asleep and did not awaken for five hours. After four days the pain and inflammation had disappeared, but the testis remained large, indurate and tender to the touch. I then strapped the testis with adhesive straps for a week, at the end of which the swelling and induration had disappeared, and the testis was a trifle larger than its fellow.—*Med. Tribune.*

**Burn Successfully Treated by Bicarbonate of Soda.**—This case is rather an unusual one, the burn having occurred under very unfavorable circumstances, as the following statement will show :

On the 28th of March, 1878, I delivered Mrs. —— of an apparently healthy female child, whose father I had treated for tertiary syphilis a few months before its birth. I was called to see the child two months after its birth, and found it afflicted with congenital syphilis—suffering with the following symptoms, viz. : hoarse voice, discharge from the nostrils, copper-colored blotches or ulcers, especially about the anus and pudenda, and somewhat emaciated. The usual remedies for syphilis were employed, and in a few months the little unfortunate seemed to be fully restored to health.

During the month of August, 1879, I was called to see the same child, and found it suffering with cholera infantum, from the effects of which it had scarcely recovered, when it received the following burns, occurring on the 21st of October : Its mother, in attempting to lift a vessel of boiling water from the stove, unfortunately upset it, pouring the entire contents upon the child, fearfully scalding one side of the body, including the head and arm. Care was taken in removing the clothes; hence the cuticle was broken in but few places.

Classifying the burn as one of the second degree, and considering the unfortunate circumstances that had attended the child from its birth, my prognosis was, of course, unfavorable. However, I adopted the following treatment: I gave opium, gr. ¼, repeated every two or three hours, to relieve the intense pain; forbade anything but a milk diet; opened the vesicles, clipping the larger ones with scissors, and pricking the smaller ones with a needle. Locally, I applied a solution of bicarbonate of soda, upon cloths, keeping them constantly saturated with the solution; the applications were renewed every four hours. This treatment was kept up for twenty-four hours, at the expiration of which time, the soda was dispensed with—the burns being in a healthy condition, and inflammation having entirely subsided, and there was no discharge and no smell.

Having seen several cases reported wherein soda was used under similar circumstances, I have been reminded to write this.—WM. B. CONWAY, M.D., in *Virginia Medical Monthly*.

**Treatment of Strychnia Poisoning.**—In a case of strychnia poisoning in a girl of 16, Rivine, a German practitioner, has found that 40 grains of potassium bromide, and 10,20 grains of chloral hydrate were of great use, whilst 120 grains of potassium bromide or 40 grains of hydrate of chloral are necessary by themselves. He therefore recommends the combination of the two remedies in the treatment of cases of poisoning by strychnia. To test this theory, his pupil, Hessler, has carried out certain experiments on rabbits. For this purpose, Hessler has investigated the mode of action of potassium bromide on the sleep produced by chloral hydrate. He finds that the simultaneous administration of the former drug does not appreciably prolong this sleep. That sensation and reflex irritability are never completely abolished; that the danger of a fatal termination would not be diminished in narcosis produced by the administration of four-fifths of the minimum lethal dose of chloral hydrate with the addition of potassium bromide in a small dose (about one-third of the lethal amount). In ten cases of the minimal lethal doses of strychnia the method advocated by Rivine was not of more use than the simple chloral treatment; by both methods the tetanus which threatened life was allayed, and consequently life was prolonged. The duration and intensity of the attack were lessened to the greatest extent when the chloral mixture was given. When the minimal lethal dose of chloral hydrate was not exceeded, a sudden diminution in the frequency of respiration, and death from chloral poisoning occasionally occurred. The ultimate conclusion to which the experiments have led is that the simple treatment of acute strychnia poisoning by means of chloral hydrate is decidedly superior to the combined method proposed by Rivine.—*Practitioner.*

**Was the Child Born Premature or at Full Term?**—This question was asked me by an attorney, to be answered for a jury's edification. My answer was that the child was born premature.

Upon what evidence do you found such a judgment?

My answer was as follows:

1. From the small size of the child—four to five pounds.

2. The nails were poorly developed, and there was but little or no hair on the head.

3. The fontanelles and sutures were uncommonly large, indicating an imperfect development of the cranial bones.

4. The dark or cyanosed color of child.

5. The presence of the membrana pupillaris, which, according to Cazeaux, Playfair, Churchill, and others, disappears in the latter part of the seventh or during the eighth month.

6. The unusual amount of vernix caseosa, which is said by Mad. Bouvin to be more abundant on premature children, and nearly always absent when parturition is delayed.

This latter statement is according to my observation, indicating that it begins to be absorbed as gestation is completed or overruns its allotted time.

7. The presentation, which was a *breach*, Faegele contends that preternatural presentations are more frequent in premature labors than when gestation has been completed.

No one of the above signs would be pathognomonic, but, taken as a whole, afford evidence, I think, sufficient to substantiate me in my diagnosis.

This was in a case of bastardy before the Circuit Court, and I had delivered the girl who brought the suit. The above were simply the facts in the case, as I observed them at the birth of the child. Her evidence (the history of the case) of course could not be taken into consideration by me in forming my judgment of the child's prematurity. It is rather difficult to explain to the laity the difference between a seven or eight months' child and one at full term; and it is sometimes not easy, especially without the history of the case, for the accoucheur himself to determine. The reasons given above seemed to satisfy the jury that the child was born "before its time," as the case turned upon that point, and the verdict was in the girl's favor.—A. G. HOBBS, M.D., in *Louisville Medical News*.

**The Cold Bath in Infantile Convulsions.**—In my own experience eighty per cent. of all cases of convulsions in children occur during fever, and I believe are nearly always caused by the elevation of temperature alone. The ordinary treatment of such cases is unsatisfactory; chloroform, first recommended by Sir James Simpson, will control the spasms, but in many cases these occur in such rapid succession that no intermission can be perceived; they continue whenever the anæsthetic is stopped, and our only recourse is to continue its administration until the fever yields to medicine or subsides spontaneously. I have followed out this plan of treatment in many cases, often successfully, and frequently not so.

I have notes of four fatal cases in which the inhalation of chloroform was continued from six to thirty hours. The administration of medicine in these cases is always difficult, sometimes impossible, and is generally attempted with risk to the already weakened heart. This is true of bromide of potassium, chloral, veratrum, aconite, etc., while quinine acts too slowly to be depended upon in any severe case; warm or hot baths are sometimes useful, when, by inducing perspiration, they reduce the temperature, but every medical man knows that they often fail to arrest the convulsions.

The cold bath fails so seldom that it may be considered a specific.

The spasms will frequently continue until the temperature has been reduced to 98½°, but at this point they are almost invariably arrested. Several years' experience with this plan of treatment has inspired me with the strongest confidence in its usefulness, and yet a desire not to have its value over-estimated compels me to admit that there are cases in which convulsions will return or continue notwithstanding the reduction of temperaiure, but such cases are rare, and probably are complicated by organic lesions, as tubercular meningitis.—*Canada Medical Record.*

**Sulphite of Soda in Malarial Fevers.**—Dr. L. Austin Porter says, in the Michigan Medical News:

I have been using sulphite of soda in many of the malarial fevers, which have been quite frequent in this neighborhood this fall. I find the results quite satisfactory, not a grain of quinine being necessary. It has appeared to me that cases thus treated are less subject to a relapse; the career of fever is cut perceptibly short, and convalescence immediately begins. I take the tongue of the patient as my guide board for the administration of this salt. When I find it presenting a broad, flabby appearance, with pale texture, and covered either with a pasty white, yellow, or brownish coat, with bad taste in mouth and fullness of stomach, sulphite of soda is my remedy; and with these signs I regard it as almost an infallible cure. But if the condition of the tongue be different from that here described, it will do more harm than good. It is absurd to administer it to a patient whose tongue is red, red-edged with fur in centre, or even covered with fur entire, while the body of it is red, or in which red papillæ are projecting up above the coating. There is nothing that will clean off the tongue quicker than this salt.

In properly selected cases it acts as a sedative, nervine, sudorific, and anodyne, bringing down the quick pulse, lessening irritability, cooling the burning skin, and soothing the aching body. My mode of giving this remedy is to use about sixty grains, divided into ten powders; one powder given every two hours, in mucilage, albumen or slippery elm tea.—*Medical and Surgical Reporter.*

**A New Rhinoplastic Operation.**—The existence of popular interest in rhinoplasty has recently been shown by a long article in a leading daily journal, describing an operation of this kind at Bellevue Hospital. The patient, a healthy young man, had lost his nose from a lupoid affection two or three years ago. The disease, having destroyed the nose, ceased to progress. The contraction of the cicatrices, however, produced ectropion in both lids. After waiting for over a year, as the disease did not show itself again, plastic operations for the relief of the ectropion were successfully performed by Dr. Sabine. These operations were followed, a short time ago, by one for the restoration of the nose, the middie finger of the left hand being used to supply the needed tissue. The nail of this finger was first removed, and its matrix cauterized. Then, at a subsequent period, the patient being etherized, an incision was made along the palmar surface of the finger, and the skin dissected back on either side. These flaps were united by fine sutures to the sides of the nasal openings. During the operation the patient was nearly strangled by the blood which poured through the nares into his larynx. Laryngotomy was instantly performed, and relief thus given. The operation was then finished, and the hand firmly bound to the face by plaster-of-Paris bandages. The patient has, we believe, since been doing well. As union takes place, the digital arteries will be tied, and eventually the finger amputated. Such further operations as are necessary for securing greater symmetry and beauty to the new organ will be performed. —*Medical Record.*

**How to Cure Fits of Sneezing.**—Surgeon S. Messenger Bradley writes to British Medical Journal :

"During the recent rapid changes of temperature I caught a severe cold in my head, accompanied by almost incessant sneezing. My unfortunate nose gave me no rest. The slightest impact of cold air, or passing from the outside air into a warm room, equally brought on a fit of sneezing. In vain I snuffed camphor and pulsatilla; the light catarrh still triumphed over me. At length I resolved to see what the maintenance of an uniform temperature would do toward diminishing the irritability of my Schneiderian membrane, and accordingly I plugged my nostrils with cotton wool. The effect was instantaneous; I sneezed no more. Again and again I tested the efficacy of this simple remedy, always with the same result. However near I was to a sneeze, the introduction of the pledgets stopped it *sur le champ*. Nor was there any inconvenience from their presence, making them sufficiently firm not to tickle, and yet leaving them sufficiently loose to easily breathe through. This is really worth knowing, for incessant sneezing is among the greatest of smaller ills, and it seems only a rational conclusion to hope that in this simple plan we may have the most efficient remedy against one of the most distressing symptoms of hay fever."—*Louisville Med. News.*

**Evidences that Dead Infants Were Born Alive.**—At the conclusion of a close study of this subject, Dr. W. S. Abbott states, in the Boston Medical and Surgical Journal, that the medical examiner may infer that a child has lived during and after its birth, from the following signs :

1. When the diaphragm reaches only to the fifth intercostal space.
2. When the lungs more or less completely fill the thorax.
3. When the ground color of the lungs is broken by sinular marblings.
4. When, by careful experiment, the lungs are found to be capable of floating.
5. When a bloody froth exudes from the cut surfaces of the lung on slight pressure.
6. When the air cells were visible to the naked eye.

These proofs, complete as they are, may be strengthened by the cicatrization of the umbilicus, the scaling of the epidermis, the closure of the fœtal ducts, the size of the osseous nucleus of the inferior femoral epiphysis, the existence of milk, sugar, starch, or medicines in the stomach, determined by the appropriate chemical tests, and by the presence of fecal matter other than meconium in the lower intestines. —*Medical and Surgical Reporter.*

**Cause of Ague.**—In two recent numbers (July and October 15th) *Archiv fur experimentelle Pathologie*, Professors Klebs and Tomasi-Crudeli report the particulars of a research recently conducted by them at Rome with regard to the essential cause of the ague which is endemic about that city; and, so far as can be judged from that account, the doctrine that ague-posion is a microphyte of malarious soil is no longer a mere matter of suspicion (as my text expresses it), but is a matter of experimental certainty. The investigators nameed declare that they can isolate from malarious soils and their atmosphere definite microphytic forms capable of separate cultivation; and that, when successive generations of this "baccillus malariæ" (as they name it) have been cultivated in successive quantities of an indifferent fluid, subcutaneous inculation of rabbits with any final fluid in which the *baccilus* is germinating will give ague to the subjects of the experiment. The argument of my text (in the section relating to the natural history of contagia) may suggest how extremely interesting it would be to ascertain whether, when animals are under influence of the *baccilus malariæ*, the fever can be propagated from them to healthy animals by means of small fransfusions of blood.—*British Medical Journal.*

**Poisonous Vinegar.**—The editor of the Boston Journal of Chemistry says:

Vinegar sold in the markets is so generally of a factitious character that it is better to discard its use, unless it can be procured from known honest sources of supply. The cheap vinegar thrown into the market by vinegar makers does not cost over two cents a gallon; some of them are but little better than dilute sulphuric acid. Pickles! If any one wishes to eat these destestable and unwholesome substances, it is better to procure the vegetables and good cider vinegar and make them at home. The oleomargarine manufacture ought to be prohibited by State legislation. Any compound made up of common beef tallow, and so colored and scented as to resemble butter, is a fraud; and most of the consumers, who are among the poorer classes, are defrauded in its purchase. The best specimens which have come under our notice are repulsive, greasy compounds.

**Morphia Tartrate.**—Morphia tartrate has been recommended by Erskine Stuart as a morphia salt particularly suitable for hypodermic injections, because more concentrated solutions can be obtained of it than of the muriate or acetate. It is very soluble in water and alcohol, forms neutral, wart-like crystals consisting of needles, and is made by dissolving ten grams crystallized morphia and 2.5 grams (or sufficient) tartaric acid in 40 grams hot distilled water, and evaporating in a moderately warm place.—*Pharm. Centralb.*

**New Method in Breech Presentation.**—Dr. Underhill, in the British Medical Journal, describes a process by which he has succeeded in difficult breech cases, after the failure of the ordinary procedures. He inserts the index and middle fingers of one hand behind the child's sacrum, and hooks them, one over each hip. He says it is very easily done.—*Pacific Med. and Surg. Journ.*

**Menthol as an Antiseptic.**—(Dr. H. Hager.) Japanese or Chinese oil of peppermint contains a large amount of stearopten (menthol), so that it forms a solid mass at ordinary temperature. This solid portion of oil of peppermint is likely to become an article much in demand, for, according to Duncan's statements, it has antiseptic powers equal to those of thymol. In some wholesale German price-lists it is quoted as Oleum Menthæ Japonicum Crystallisatum, price 62 marks (nearly $16) the kilo.—*Pharm. Centralb.*, 394.

**Sclerotic Acid.**—Speaking of this substance the Medical Times and Gazette says: It has the advantage of remaining indefinitely without loss of strength, if only kept in a dry place and undissolved. Its sodium salt Nitikin considers the best preparation for internal use in the human subject. Subcutaneous injection of either drug causes a "sharp biting" pain, which passes off in a few minutes. Von Zeimssen claims for Sclerotic Acid over Ergotin that the former causes no inflammation at the seat of puncture.

**Inaction of the Bladder.**—Dr. Harrison (in *Lancet*) says:
Of the medicines that I have found most useful in restoring the tone of the bladder, I would mention the ergot of rye, which I generally give in doses of twenty to thirty minims of the fluid extract in cinnamon-water. Of its use, further experience only strengthens the good opinion of it I have elsewhere expressed in the treatment of this complication of prostatic enlargement.

**On the Use of Carlsbad Water in Diabetes Mellitus.**—Dr. Mayer, of Carlsbad, concludes a paper on this subject with the following resume:

I. Diabetes mellitus is an incurable disease.

II. The Carlsbad cure, in a large number of cases, relieves the most troublesome symptoms, improves the general condition of the patients, and prolongs life.

**Carnauba Root.**—This recent introduction to the list of therapeutic agents appears, from the very scant literature which we have been able to secure on the subject, to merit a thorough trial. It is classed under the head of vegetable alteratives, and its use in Brazil, to which country it is indigenous, has made it very popular in the class of cases in which sarsaparilla, stillingia, etc., are employed with us.—*New Press.*

**Cayaponin.**—The alkaloid of Cayaponia Globulosa, a cucurbitaceous plant of Brazil, is a powerful purgative in doses of six milligrams (1-10 grain) without causing griping.—*Rep. de Ph., and Ph. Zeit, f. Russt.*

SEVERAL cases are reported in England in which scarlet fever, measles, and other infectious diseases, have been spread by means of domestic animals, as cats and dogs.

